# Crystal structure of 2-oxo-2*H*-chromen-7-yl tri­fluoro­methane­sulfonate

**DOI:** 10.1107/S205698902600071X

**Published:** 2026-01-29

**Authors:** Navneet Goyal, Rafia Rashid, Xiaodong Zhang, Abha Verma, Rami A. Al-Horani, James P. Donahue

**Affiliations:** a Department of Chemistry Xavier University of Louisiana 1 Drexel Drive New Orleans LA 70125 USA; b Department of Chemistry, Tulane University, 6400 Freret Street, New Orleans, Louisiana 70118-5698, USA; c Division of Basic Pharmaceutical Sciences College of Pharmacy Xavier University of Louisiana 1 Drexel Drive New Orleans LA 70125 USA; Texas A & M University, USA

**Keywords:** crystal structure, coumarin, tri­fluoro­methane­sulfonate-substituted

## Abstract

Crystalline 2-oxo-2*H*-chromen-7-yl tri­fluoro­methane­sulfonate, C_10_H_5_F_3_O_5_S, forms in the monoclinic space group *C*2/*c* as pseudoplanar dyads about an inversion center, with close packing assisted by inter­molecular C—H⋯O contacts of 2.38 Å. These dyads are arranged into a staircase pattern where the direction and pitch of the staircase are aligned with the *b* axis. Between adjacent stacks, mol­ecules are related by a 2_1_ axis and held in place by a herringbone-like network of additional inter­molecular C—H⋯O contacts of 2.35 Å.

## Abstract 2

The title compound, 2-oxo-2*H*-chromen-7-yl-tri­fluoro­methane­sulfonate, crystallizes as closely associated pairs across an inversion center, these dyads then forming steps in a staircase packing arrangement in the direction of the *b* axis. Efficient crystal packing is assisted by a dense network of C—H⋯O inter­molecular contacts.

## 1. Chemical context

Hy­droxy coumarins, such as 7-hy­droxy­coumarin (umbelliferone), are derivatives of common coumarin that posses hydroxyl groups, which enhance their solubility and biological activity (Kornicka *et al.*, 2023 ▸). These compounds find use in the detection of toxins such as cyanide (Eapen *et al.*, 2025 ▸), Hg^2+^ (Li *et al.*, 2020 ▸), and Pb^2+^ (Sharma & Gulati, 2021 ▸). They exhibit a broad spectrum of pharmacological properties, including anti­oxidant, anti­coagulant, and anti­microbial activity, making them valuable in pharmaceutical research and applications (Kornicka *et al.*, 2023 ▸; Lee *et al.*, 2008 ▸).
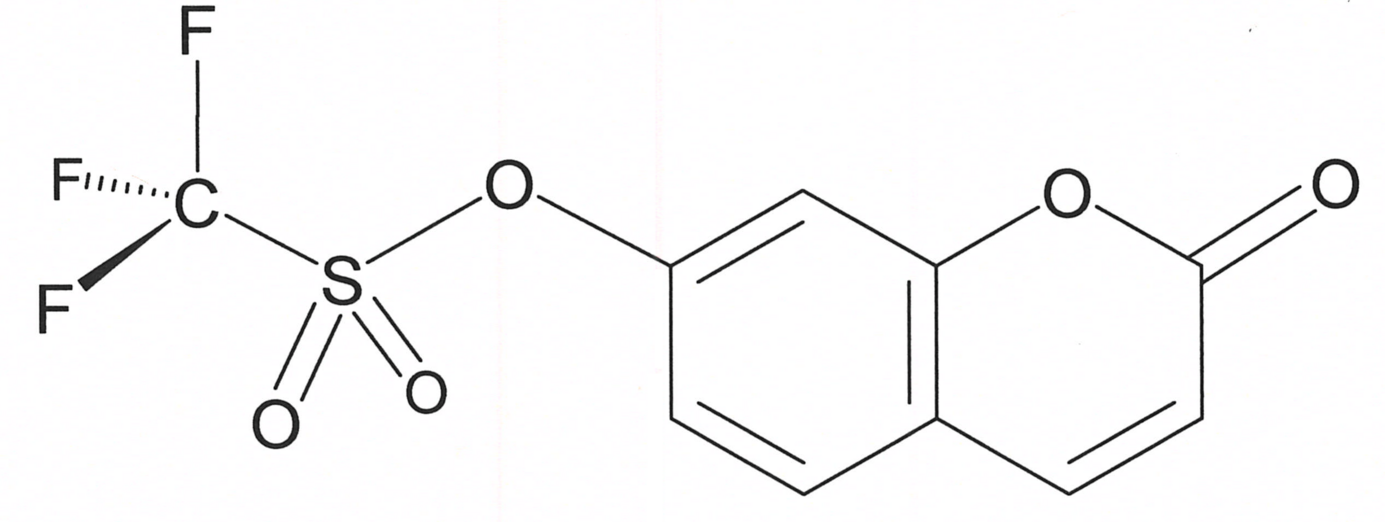


In synthetic organic chemistry, the hy­droxy group of 7-hy­droxy­coumarin can be converted into an enhanced leaving group by reaction with triflic anhydride, thereby affording 2-oxo-2*H*-chromen-7-yl-tri­fluoro­methane­sulfonate, **I**. This triflate derivative is a highly activated electrophile, which can undergo cross-coupling (Zhang *et al.*, 2007 ▸) or nucleophilic substitution to install diverse substituents on the coumarin core (Grimm & Lavis, 2011 ▸), expanding the structural and functional diversity for pharmaceutical and agrochemical exploration and for development of fluoro­phore-based imaging agents (Otsuka *et al.*, 2016 ▸; López-Corrales *et al.*, 2023 ▸).

## 2. Structural commentary

**Figure 1:**
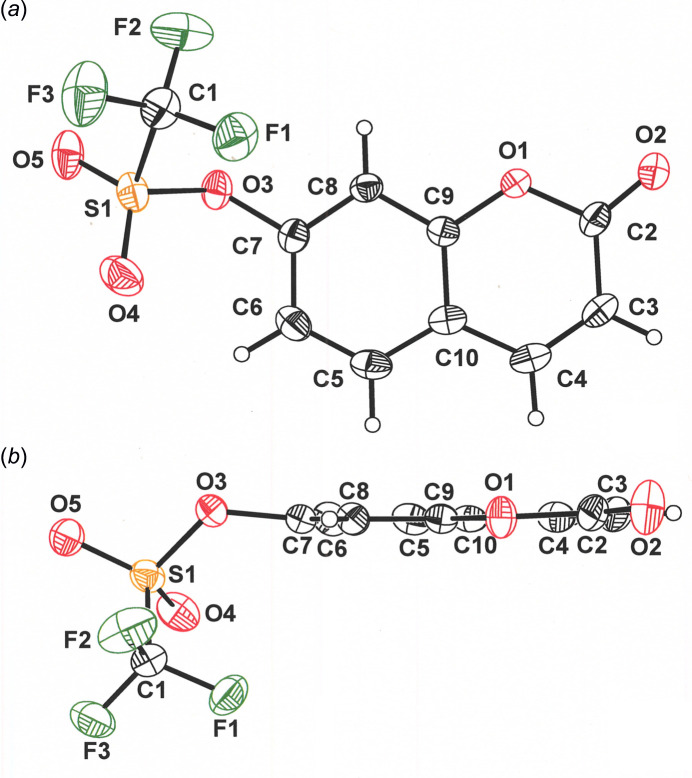
(*a*) Displacement ellipsoid plot (50% probability) of 2-oxo-2*H*-chromen-7-yl-tri­fluoro­methane­sulfonate, **I**, with complete atom labeling. (*b*) View of **I** along the edge of the coumarin plane. This perspective is from a position that is rotated by 90° from that shown in (*a*). Displacement ellipsoids are presented at the 50% probability level.

Fig. 1 ▸(*a*) presents a view of 2-oxo-2*H*-chromen-7-yl-tri­fluoro­methane­sulfonate that is orthogonal to the coumarin ring system, with atom labeling in accord with the IUPAC numbering system (Annunziata *et al.*, 2020 ▸). The eleven atoms of the coumarin ring system are planar with a mean deviation of 0.021 Å. The maximum out-of-plane deviation is shown by O1 [0.039 (1) Å], as it is the only exocyclic atom of the parent structure. As seen in Fig. 1 ▸(*b*), O3 is bent slightly, but discernibly [0.173 (1) Å], to the upper face of the coumarin plane, while the triflate substituent as a whole is oriented downward toward the opposite (bottom) face. The C7—O3—S1—C1 torsion angle is 81.57 (15)°. The inter­atomic distances within the coumarin core of **I** are the same, within experimental resolution, as those observed in *P*2_1_ (Zhang *et al.*, 2021 ▸) and *Pca*2_1_ (Waddell *et al.*, 2024 ▸) polymorphs of coumarin itself.

## 3. Supra­molecular features

**Figure 2:**
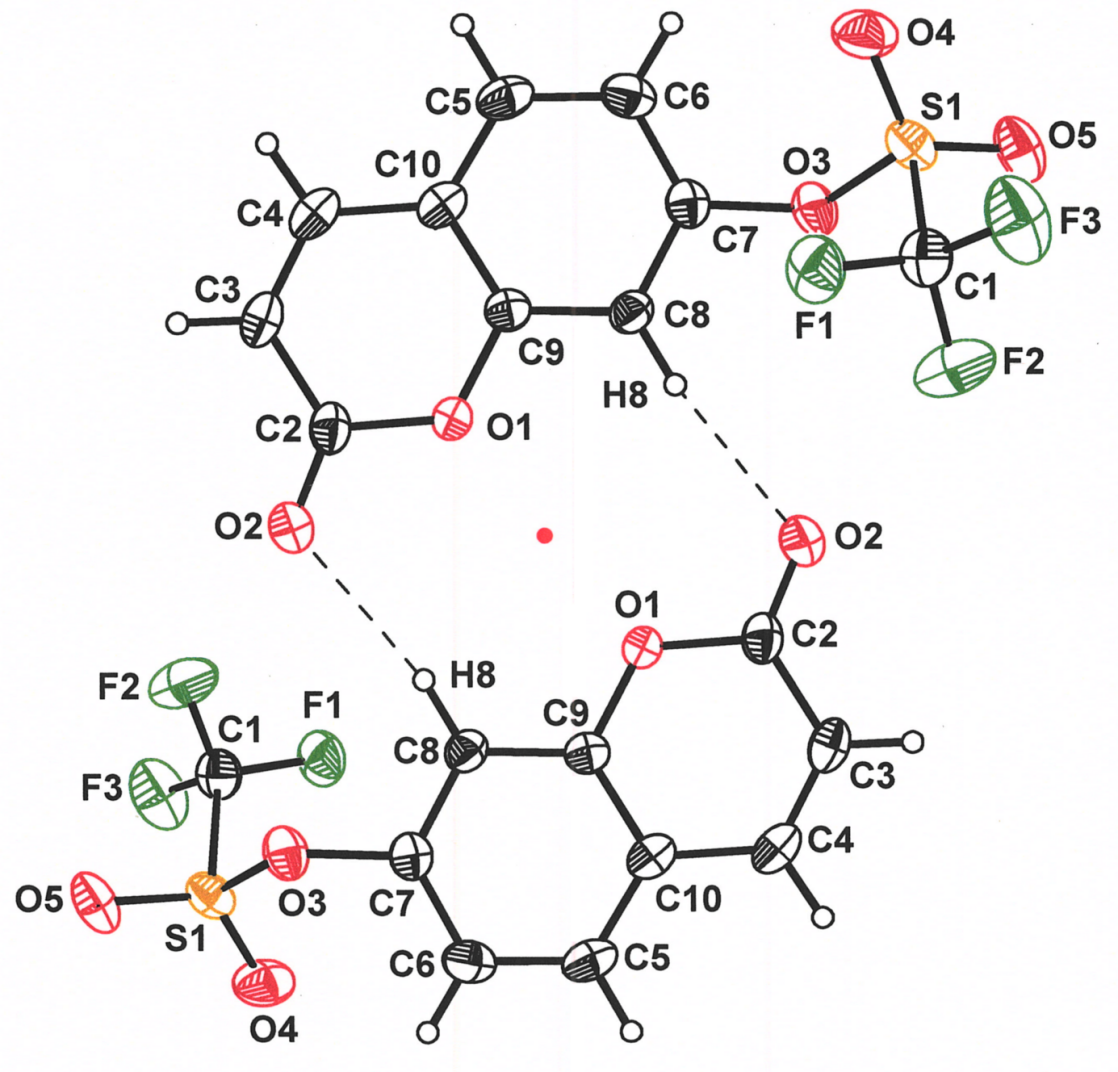
Pseudoplanar dyads of **I** formed by pairwise C8—H8⋯O2 inter­actions across an inversion center. The position of the inversion center is marked with a red point. Displacement ellipsoids are shown at 50%.

**Figure 3:**
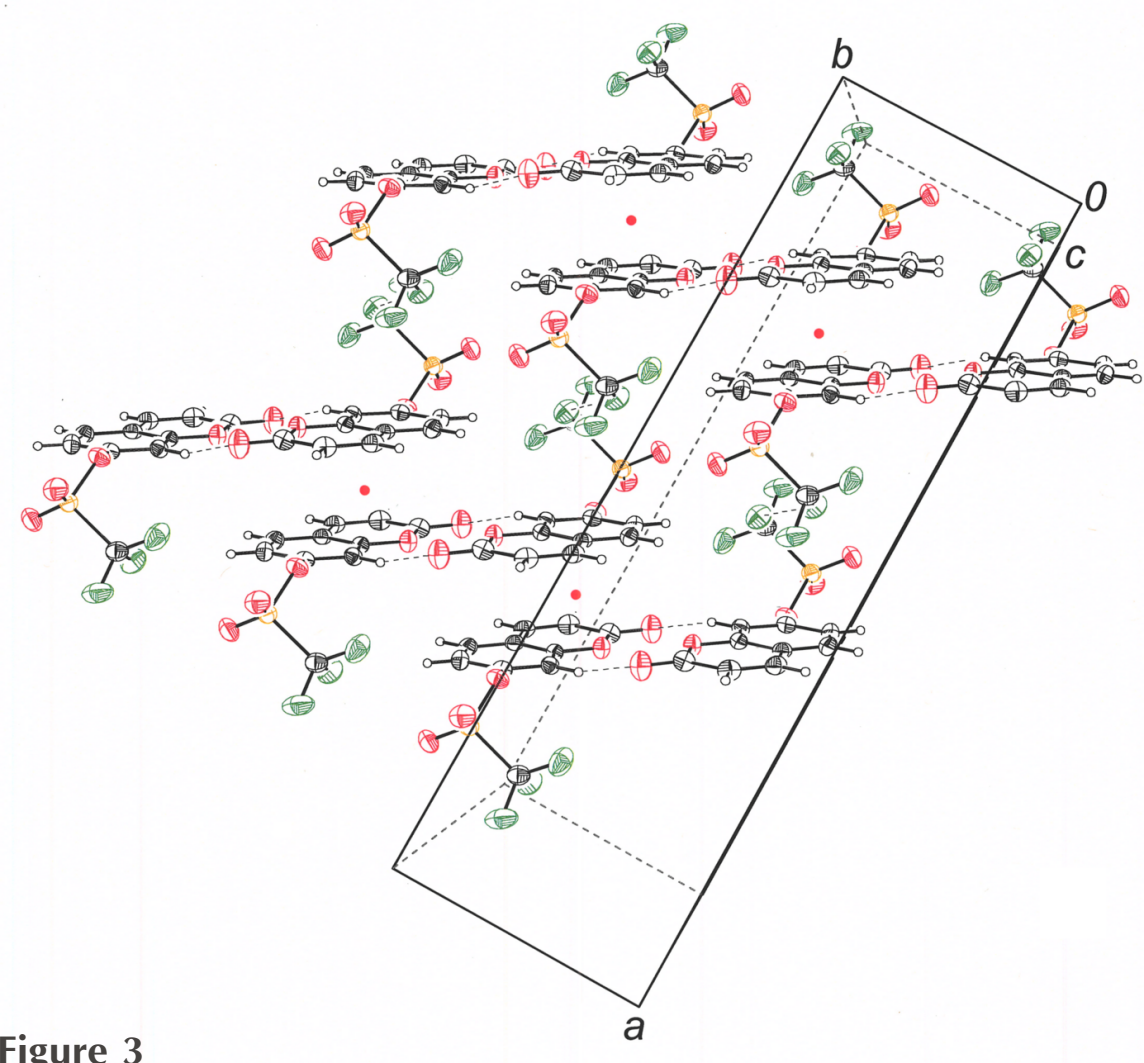
View down the *c* axis of the cell showing the staircase-like arrangement of dyads of **I** along the *b* axis of the cell. Displacement ellipsoids are drawn at the 50% probability level. Red points designate the positions of inversion centers that relate each step of the staircase to its symmetry partners above and below. Displacement ellipsoids are drawn at the 50% level.

The extended packing pattern of 2-oxo-2*H*-chromen-7-yl-tri­fluoro­methane­sulfonate mol­ecules begins with pseudo-coplanar pairs disposed around an inversion center and held in place, in part, via C8—H8⋯O2 close contacts of 2.38 Å (Table 1 ▸, Fig. 2 ▸). These dyads are arranged in a staircase-like fashion that places the top face of the coumarin ring system of one dyad member into a π–π stacking inter­action with the step above, while the bottom face of its centrosymmetric partner is similarly related to the dyad that forms the step below (Fig. 3 ▸). The π–π stacks are also formed around inversion centers, marked as red points in Fig. 3 ▸, a consequence of which is that the coumarin rings of different steps approach one another in a tail-to-tail manner but with a sideways slip that places carbon 9 of one mol­ecule above carbon 9 of its partner at a separation of 3.443 (2) Å. This distance is well within the 3.3-3.8 Å that is regarded as typical of offset parallel planar aromatic ring inter­actions (Janiak, 2000 ▸). The stair-like formations of dyads lie in the *ab* plane of the unit cell, with the *b* axis defining both the direction and the pitch of the staircase (Fig. 3 ▸).

**Figure 4:**
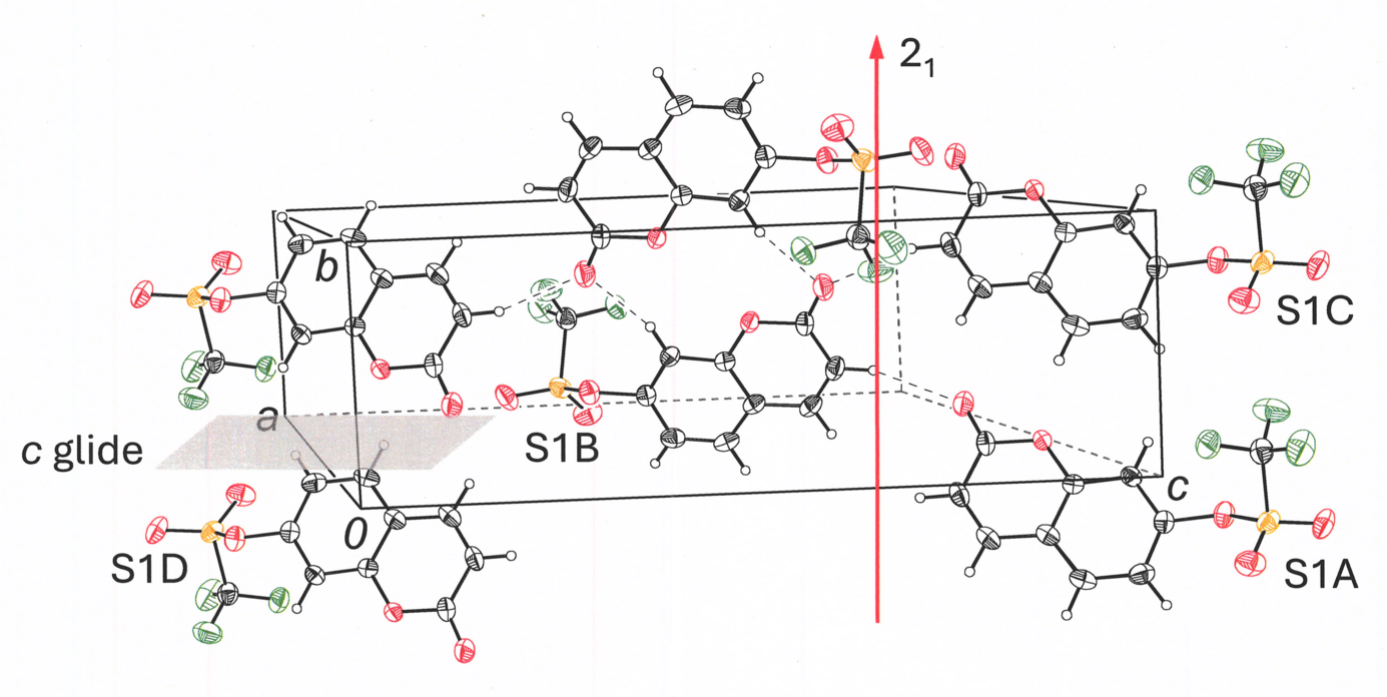
Partial packing of the unit cell for **I** showing mol­ecules related by 2_1_ symmetry and by the *c* glide operation. Displacement ellipsoids are shown at 50%.

**Figure 5:**
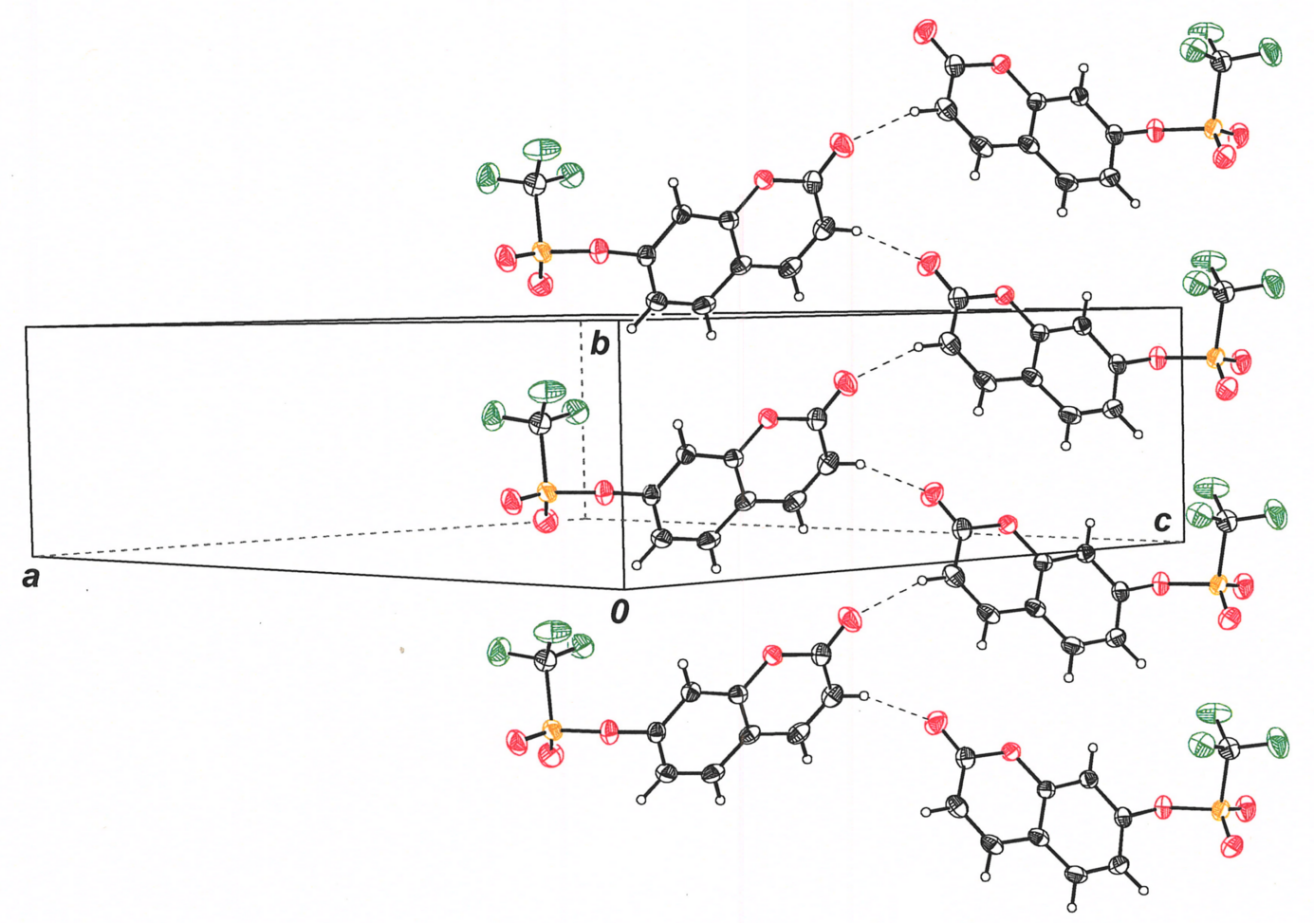
View approximately along the *ac* face diagonal of the cell showing adjacent mol­ecular stacks in the direction of the *b* axis. Inter­molecular C3—H3⋯O2 close contacts are illustrated with dashed lines. Displacement ellipsoids are drawn at the 50% level.

**Figure 6:**
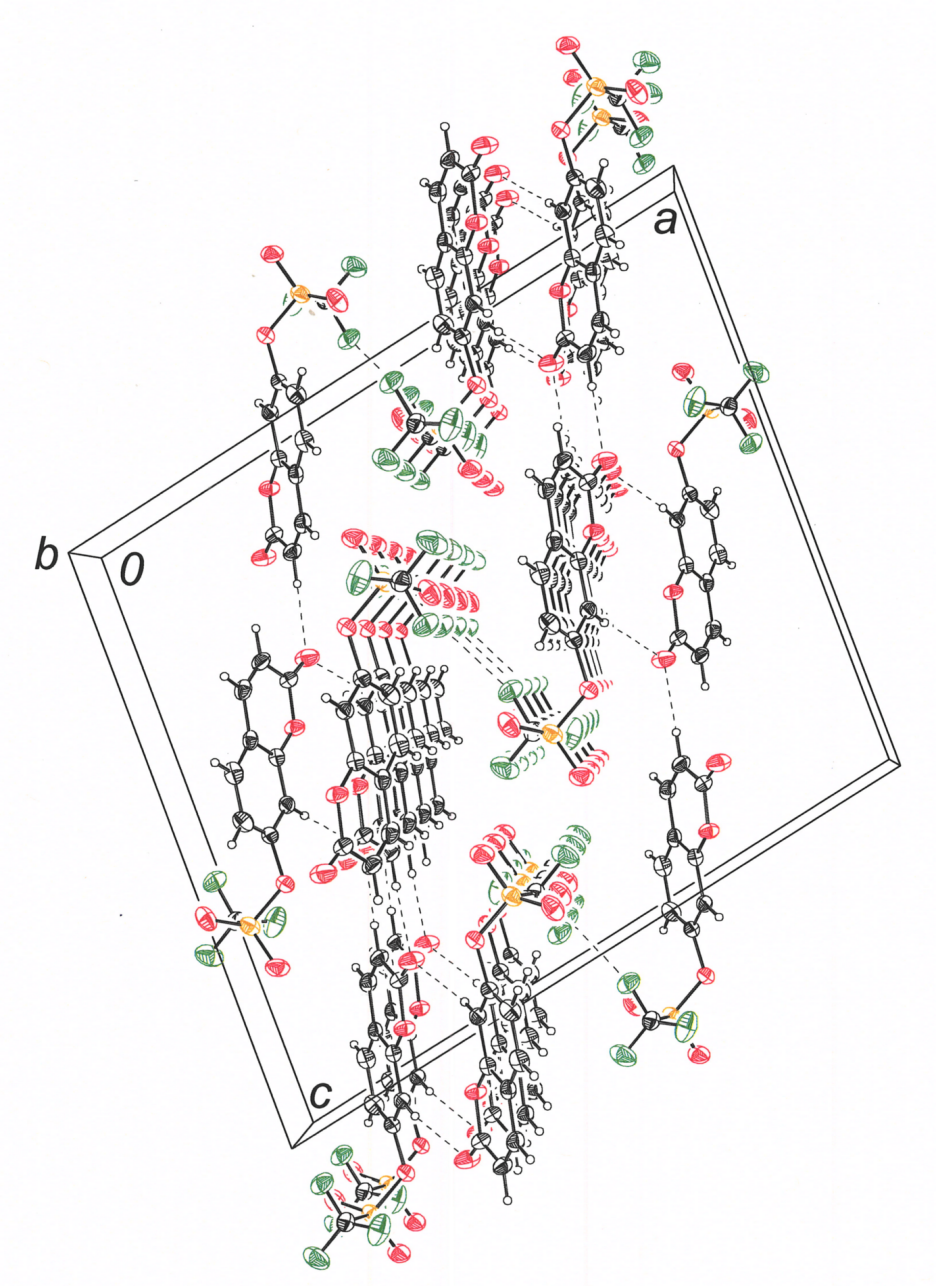
View down the *b* axis of the cell showing eclipsed columnar stacks of **I**. Displacement ellipsoids are shown at 50%.

Between the staircase-like stacks of mol­ecules on the *ab* faces of the cell lie other stacks to which they are related by 2_1_ axes. Fig. 4 ▸ illustrates how the mol­ecule bearing the S1B label is related by a 2_1_ axis in red to the mol­ecules with the S1A and S1C labels. Similarly, this same mol­ecule is related to the mol­ecule with the S1D label by a *c* glide plane (in gray), which is parallel to the *ac* plane and orthogonal to the *b* axis (Fig. 4 ▸). A consequence of the positioning of 2_1_ axis between adjacent stacks is that the coumarin mol­ecular planes in adjoining stacks have the same angular disposition of 26° relative to the stacking axis but in opposite directions (Fig. 5 ▸). Thus, when these stacks are considered from the perspective along the stacking axis, mol­ecules similarly eclipse one another without giving clear evidence of the alternating angle of cant between them (Fig. 6 ▸). Close contacts of 2.35 Å between C3–H3⋯O2 form the predominant inter­stack inter­actions (Table 1 ▸, Fig. 5 ▸). Although F1⋯F1 inter­molecular contacts of 2.863 (2) Å do occur (not illustrated), which is less than the 3.00 Å sum of the crystallographic van der Waals radii (Batsanov, 2001 ▸), the 139.97° C—F⋯F angle places this inter­action into the Type I category of nonstabilizing F⋯F inter­actions that is driven by the larger packing pattern (Reichenbächer *et al.*, 2005 ▸).

## 4. Database survey

Related mol­ecules that have been identified structurally by X-ray crystallography are 4-tri­fluoro­methyl-7-tri­fluoro­methane­sulfonato-2*H*-chromen-2-one (Qin *et al.*, 2025 ▸), 6,7-bis­(tri­fluoro­methane­sulphonato)-4-methyl-2*H*-chromen-2-one (Hamdy *et al.*, 2016 ▸), 7-(4-meth­oxy­phen­yl)-4-methyl-6- tri­fluoro­methane­sulphonato-2*H*-chromen-2-one (Hamdy *et al.*, 2016 ▸), and 6,7-bis­(tri­fluoro­methane­sulfonato)-3-bromo-4-methyl-2-oxo-2*H*-chromene (Hamdy *et al.*, 2016 ▸).

## 5. Synthesis and crystallization

The starting material, 7-hy­droxy­coumarin (0.500 g, 3.08 mmol) was dissolved in dry THF (25 ml) under an N_2_ atmosphere and cooled to 273 K. Tri­ethyl­amine (1.1 ml, 7.70 mmol) was added to the reaction mixture. After stirring for 15 min at 273 K, triflic anhydride (0.57 ml, 3.39 mmol) was added dropwise. The reaction mixture was stirred at ambient temperature for 2 h, and progress of the reaction was monitored using thin-layer chromatography (TLC). The reaction mixture was extracted with EtOAc (50 ml) and H_2_O (25 ml). The organic layer was separated and dried over anyhdrous sodium sulfate. The solvent was evaporated under vacuum, and the crude product was purified by flash column chromatography on silica gel using 1:3 EtOAc:hexa­nes (*v*/*v*) as the eluant to yield the target compound as a white solid (0.810 g, 90% yield). Crystals were obtained by slow cooling of a warm solution in 2:1 EtOAc:hexa­nes (*v*/*v*). ^1^H NMR (300 MHz, δ, p.p.m. in CDCl_3_): 6.50 (*d*, *J* = 9.6 Hz, 1 H), 7.22–7.26 (*m*, 1 H), 7.27–7.30 (*m*, 1 H), 7.61 (*d*, *J* = 8.4 Hz, 1 H), 7.74 (*d*, *J* = 9.6 Hz, 1 H). ^13^C NMR (75 MHz, δ, p.p.m. in CDCl_3_): 110.5, 117.7, 177.8, 188.8, 120.3, 129.5, 142.1, 150.8, 154.6, 159.3.

## 6. Refinement

Crystal data, data collection and structure refinement details are summarized in Table 2 ▸. Hydrogen atoms were added in calculated positions and refined with isotropic displacement parameters that were approximately 1.2 times (for aromatic C—H) those of the carbon atoms to which they were attached. The C—H distance assumed was 0.95 Å.

## Supplementary Material

Crystal structure: contains datablock(s) I, global. DOI: 10.1107/S205698902600071X/jy2068sup1.cif

Structure factors: contains datablock(s) I. DOI: 10.1107/S205698902600071X/jy2068Isup2.hkl

CCDC reference: 2525434

Additional supporting information:  crystallographic information; 3D view; checkCIF report

## Figures and Tables

**Table 1:** Hydrogen-bond geometry (Å, °)

*D*—H⋯*A*	*D*—H	H⋯*A*	*D*⋯*A*	*D*—H⋯*A*
C3—H3⋯O2^i^	0.95	2.35	3.267 (2)	162
C8—H8⋯O2^ii^	0.95	2.38	3.293 (2)	162

Symmetry codes: (i) 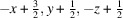; (ii) 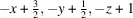.

**Table 2:** Experimental details

Crystal data	
Chemical formula	C_10_H_5_F_3_O_5_S
*M*_r_	294.20
Crystal system, space group	Monoclinic, *C*2/*c*
Temperature (K)	150
*a*, *b*, *c* (Å)	20.4767 (9), 6.0828 (3), 18.2181 (8)
β (°)	102.137 (2)
*V* (Å^3^)	2218.45 (18)
*Z*	8
Radiation type	Mo *K*α
μ (mm^−1^)	0.35
Crystal size (mm)	0.17 × 0.06 × 0.03

Data collection	
Diffractometer	Bruker D8
Absorption correction	Multi-scan (*SADABS*; Krause *et al.*, 2015 ▸)
*T*_min_, *T*_max_	0.927, 0.988
No. of measured, independent and observed [*I* > 2σ(*I*)] reflections	28942, 3542, 2127
*R*_int_	0.069
(sin θ/λ)_max_ (Å^−1^)	0.725

Refinement	
*R*[*F*^2^ > 2σ(*F*^2^)], *wR*(*F*^2^), *S*	0.044, 0.107, 1.04
No. of reflections	3542
No. of parameters	172
H-atom treatment	H-atom parameters constrained
Δρ_max_, Δρ_min_ (e Å^−3^)	0.35, −0.37

Computer programs: *APEX5* and *SAINT* (Bruker, 2024 ▸), *SHELXT2018/2* (Sheldrick, 2015*a* ▸), *SHELXL2019/2* (Sheldrick, 2015*b* ▸) and *SHELXTL* (Sheldrick, 2008 ▸).

## supplementary crystallographic information

### 2-Oxo-2H-chromen-7-yl trifluoromethanesulfonate Crystal data

**Table:**

C_10_H_5_F_3_O_5_S	*F*(000) = 1184
*M**_r_* = 294.20	*D*_x_ = 1.762 Mg m^−^^3^
Monoclinic, *C*2/*c*	Mo *K*α radiation, λ = 0.71073 Å
*a* = 20.4767 (9) Å	Cell parameters from 4023 reflections
*b* = 6.0828 (3) Å	θ = 3.4–25.5°
*c* = 18.2181 (8) Å	µ = 0.35 mm^−^^1^
β = 102.137 (2)°	*T* = 150 K
*V* = 2218.45 (18) Å^3^	Block, colorless
*Z* = 8	0.17 × 0.06 × 0.03 mm

### 2-Oxo-2H-chromen-7-yl trifluoromethanesulfonate Data collection

**Table:**

Bruker D8 diffractometer	3542 independent reflections
Radiation source: sealed tube	2127 reflections with *I* > 2σ(*I*)
Flat graphite monochromator	*R*_int_ = 0.069
Detector resolution: 7.391 pixels mm^-1^	θ_max_ = 31.0°, θ_min_ = 2.3°
ω and φ scans	*h* = −29→29
Absorption correction: multi-scan (*SADABS*; Krause *et al.*, 2015)	*k* = −8→8
*T*_min_ = 0.927, *T*_max_ = 0.988	*l* = −26→26
28942 measured reflections	

### 2-Oxo-2H-chromen-7-yl trifluoromethanesulfonate Refinement

**Table:**

Refinement on *F*^2^	Primary atom site location: intrinsic phasing
Least-squares matrix: full	Hydrogen site location: inferred from neighbouring sites
*R*[*F*^2^ > 2σ(*F*^2^)] = 0.044	H-atom parameters constrained
*wR*(*F*^2^) = 0.107	*w* = 1/[σ^2^(*F*_o_^2^) + (0.0376*P*)^2^ + 1.1073*P*] where *P* = (*F*_o_^2^ + 2*F*_c_^2^)/3
*S* = 1.04	(Δ/σ)_max_ = 0.001
3542 reflections	Δρ_max_ = 0.35 e Å^−^^3^
172 parameters	Δρ_min_ = −0.37 e Å^−^^3^
0 restraints	

### 2-Oxo-2H-chromen-7-yl trifluoromethanesulfonate Special details

**Table:**

Geometry. All esds (except the esd in the dihedral angle between two l.s. planes) are estimated using the full covariance matrix. The cell esds are taken into account individually in the estimation of esds in distances, angles and torsion angles; correlations between esds in cell parameters are only used when they are defined by crystal symmetry. An approximate (isotropic) treatment of cell esds is used for estimating esds involving l.s. planes.

### 2-Oxo-2H-chromen-7-yl trifluoromethanesulfonate Fractional atomic coordinates and isotropic or equivalent isotropic displacement parameters (Å^2^)

**Table:**

	*x*	*y*	*z*	*U*_iso_*/*U*_eq_	
S1	0.56456 (2)	0.72261 (8)	0.66170 (3)	0.03075 (14)	
F1	0.52714 (6)	0.3963 (2)	0.57032 (6)	0.0431 (3)	
F2	0.58390 (7)	0.3006 (2)	0.67780 (8)	0.0543 (4)	
F3	0.48224 (7)	0.4049 (2)	0.66635 (8)	0.0565 (4)	
O1	0.71324 (6)	0.4339 (2)	0.43122 (7)	0.0282 (3)	
O2	0.76131 (8)	0.2925 (2)	0.34462 (8)	0.0412 (4)	
O3	0.63257 (6)	0.7226 (2)	0.63519 (7)	0.0308 (3)	
O4	0.51608 (7)	0.8532 (2)	0.61494 (9)	0.0426 (4)	
O5	0.58160 (7)	0.7476 (2)	0.74031 (8)	0.0406 (4)	
C1	0.53785 (10)	0.4375 (3)	0.64313 (11)	0.0322 (5)	
C2	0.72999 (10)	0.4453 (3)	0.36176 (10)	0.0291 (4)	
C3	0.70878 (10)	0.6384 (3)	0.31694 (11)	0.0316 (4)	
H3	0.718411	0.649575	0.268294	0.038*	
C4	0.67597 (9)	0.8014 (3)	0.34233 (11)	0.0306 (4)	
H4	0.662584	0.926352	0.311571	0.037*	
C5	0.62832 (10)	0.9553 (3)	0.44742 (12)	0.0322 (5)	
H5	0.614123	1.084405	0.419228	0.039*	
C6	0.61648 (10)	0.9353 (3)	0.51895 (12)	0.0324 (5)	
H6	0.594656	1.048660	0.540455	0.039*	
C7	0.63747 (9)	0.7445 (3)	0.55832 (10)	0.0258 (4)	
C8	0.66927 (9)	0.5751 (3)	0.53042 (10)	0.0248 (4)	
H8	0.682938	0.446113	0.558939	0.030*	
C9	0.68042 (8)	0.6017 (3)	0.45848 (10)	0.0239 (4)	
C10	0.66065 (9)	0.7901 (3)	0.41557 (11)	0.0262 (4)	

### 2-Oxo-2H-chromen-7-yl trifluoromethanesulfonate Atomic displacement parameters (Å^2^)

**Table:**

	*U*^11^	*U*^22^	*U*^33^	*U*^12^	*U*^13^	*U*^23^
S1	0.0284 (3)	0.0301 (3)	0.0362 (3)	−0.0015 (2)	0.0125 (2)	−0.0065 (2)
F1	0.0539 (8)	0.0436 (7)	0.0294 (7)	−0.0069 (6)	0.0033 (6)	−0.0088 (6)
F2	0.0619 (9)	0.0329 (7)	0.0562 (9)	0.0004 (6)	−0.0148 (7)	0.0039 (6)
F3	0.0522 (8)	0.0631 (9)	0.0614 (9)	−0.0282 (7)	0.0284 (7)	−0.0191 (7)
O1	0.0372 (8)	0.0259 (7)	0.0233 (7)	0.0058 (6)	0.0103 (6)	0.0042 (6)
O2	0.0562 (10)	0.0418 (9)	0.0283 (8)	0.0135 (7)	0.0153 (7)	0.0012 (7)
O3	0.0243 (7)	0.0407 (8)	0.0289 (8)	−0.0015 (6)	0.0088 (6)	−0.0029 (6)
O4	0.0313 (8)	0.0391 (9)	0.0596 (11)	0.0098 (7)	0.0142 (7)	0.0059 (7)
O5	0.0439 (9)	0.0466 (9)	0.0349 (9)	−0.0097 (7)	0.0166 (7)	−0.0173 (7)
C1	0.0309 (11)	0.0362 (11)	0.0284 (11)	−0.0047 (9)	0.0034 (9)	−0.0028 (9)
C2	0.0306 (10)	0.0363 (11)	0.0198 (10)	−0.0003 (9)	0.0042 (8)	0.0008 (8)
C3	0.0342 (11)	0.0381 (11)	0.0209 (10)	−0.0031 (9)	0.0024 (8)	0.0061 (9)
C4	0.0284 (10)	0.0308 (10)	0.0298 (11)	−0.0042 (8)	−0.0003 (8)	0.0103 (8)
C5	0.0291 (10)	0.0237 (10)	0.0429 (13)	0.0000 (8)	0.0056 (9)	0.0083 (9)
C6	0.0313 (11)	0.0252 (10)	0.0424 (13)	0.0019 (8)	0.0119 (9)	−0.0014 (9)
C7	0.0223 (9)	0.0302 (10)	0.0250 (10)	−0.0028 (8)	0.0055 (8)	−0.0011 (8)
C8	0.0232 (9)	0.0259 (10)	0.0249 (10)	0.0018 (7)	0.0043 (8)	0.0044 (8)
C9	0.0212 (9)	0.0234 (9)	0.0265 (10)	−0.0002 (7)	0.0037 (7)	0.0005 (8)
C10	0.0210 (9)	0.0255 (9)	0.0298 (10)	−0.0036 (7)	0.0002 (8)	0.0056 (8)

### 2-Oxo-2H-chromen-7-yl trifluoromethanesulfonate Geometric parameters (Å, º)

**Table:**

S1—O5	1.4092 (15)	C3—H3	0.9500
S1—O4	1.4094 (15)	C4—C10	1.435 (3)
S1—O3	1.5671 (13)	C4—H4	0.9500
S1—C1	1.829 (2)	C5—C6	1.380 (3)
F1—C1	1.322 (2)	C5—C10	1.395 (3)
F2—C1	1.316 (2)	C5—H5	0.9500
F3—C1	1.310 (2)	C6—C7	1.384 (3)
O1—C9	1.372 (2)	C6—H6	0.9500
O1—C2	1.381 (2)	C7—C8	1.372 (3)
O2—C2	1.207 (2)	C8—C9	1.386 (2)
O3—C7	1.431 (2)	C8—H8	0.9500
C2—C3	1.444 (3)	C9—C10	1.398 (2)
C3—C4	1.334 (3)		
			
O5—S1—O4	123.08 (9)	C3—C4—H4	119.6
O5—S1—O3	105.48 (8)	C10—C4—H4	119.6
O4—S1—O3	111.86 (8)	C6—C5—C10	121.48 (18)
O5—S1—C1	106.92 (9)	C6—C5—H5	119.3
O4—S1—C1	106.11 (10)	C10—C5—H5	119.3
O3—S1—C1	101.16 (8)	C5—C6—C7	117.77 (18)
C9—O1—C2	121.96 (14)	C5—C6—H6	121.1
C7—O3—S1	123.46 (11)	C7—C6—H6	121.1
F3—C1—F2	109.46 (18)	C8—C7—C6	123.88 (17)
F3—C1—F1	108.86 (16)	C8—C7—O3	115.41 (16)
F2—C1—F1	108.22 (17)	C6—C7—O3	120.45 (16)
F3—C1—S1	109.27 (14)	C7—C8—C9	116.58 (17)
F2—C1—S1	110.80 (13)	C7—C8—H8	121.7
F1—C1—S1	110.20 (14)	C9—C8—H8	121.7
O2—C2—O1	116.49 (17)	O1—C9—C8	116.47 (15)
O2—C2—C3	126.38 (18)	O1—C9—C10	120.92 (16)
O1—C2—C3	117.13 (17)	C8—C9—C10	122.60 (17)
C4—C3—C2	121.47 (18)	C5—C10—C9	117.68 (17)
C4—C3—H3	119.3	C5—C10—C4	124.61 (17)
C2—C3—H3	119.3	C9—C10—C4	117.70 (17)
C3—C4—C10	120.76 (18)		
			
O5—S1—O3—C7	−167.17 (14)	C5—C6—C7—C8	0.1 (3)
O4—S1—O3—C7	−31.00 (16)	C5—C6—C7—O3	173.90 (17)
C1—S1—O3—C7	81.57 (15)	S1—O3—C7—C8	−121.98 (16)
O5—S1—C1—F3	66.41 (16)	S1—O3—C7—C6	63.7 (2)
O4—S1—C1—F3	−66.57 (16)	C6—C7—C8—C9	0.1 (3)
O3—S1—C1—F3	176.56 (13)	O3—C7—C8—C9	−173.99 (15)
O5—S1—C1—F2	−54.27 (17)	C2—O1—C9—C8	−176.98 (16)
O4—S1—C1—F2	172.75 (14)	C2—O1—C9—C10	2.0 (3)
O3—S1—C1—F2	55.87 (16)	C7—C8—C9—O1	178.91 (15)
O5—S1—C1—F1	−174.02 (13)	C7—C8—C9—C10	0.0 (3)
O4—S1—C1—F1	53.00 (16)	C6—C5—C10—C9	0.4 (3)
O3—S1—C1—F1	−63.87 (15)	C6—C5—C10—C4	−178.56 (18)
C9—O1—C2—O2	176.94 (16)	O1—C9—C10—C5	−179.14 (16)
C9—O1—C2—C3	−2.8 (2)	C8—C9—C10—C5	−0.2 (3)
O2—C2—C3—C4	−177.9 (2)	O1—C9—C10—C4	−0.1 (3)
O1—C2—C3—C4	1.7 (3)	C8—C9—C10—C4	178.84 (17)
C2—C3—C4—C10	0.1 (3)	C3—C4—C10—C5	178.07 (19)
C10—C5—C6—C7	−0.4 (3)	C3—C4—C10—C9	−0.9 (3)

### 2-Oxo-2H-chromen-7-yl trifluoromethanesulfonate Hydrogen-bond geometry (Å, º)

**Table:**

*D*—H···*A*	*D*—H	H···*A*	*D*···*A*	*D*—H···*A*
C3—H3···O2^i^	0.95	2.35	3.267 (2)	162
C8—H8···O2^ii^	0.95	2.38	3.293 (2)	162

Symmetry codes: (i) −*x*+3/2, *y*+1/2, −*z*+1/2; (ii) −*x*+3/2, −*y*+1/2, −*z*+1.
